# Bacterial Long-Chain Polyunsaturated Fatty Acids: Their Biosynthetic Genes, Functions, and Practical Use

**DOI:** 10.3390/md14050094

**Published:** 2016-05-12

**Authors:** Kiyohito Yoshida, Mikako Hashimoto, Ryuji Hori, Takumi Adachi, Hidetoshi Okuyama, Yoshitake Orikasa, Tadashi Nagamine, Satoru Shimizu, Akio Ueno, Naoki Morita

**Affiliations:** 1 Laboratory of Ecological Genetics, Section of Environmental Biology, Faculty of Environmental Earth Science, Hokkaido University, Kita-ku, Sapporo, Hokkaido 060-0810, Japan; majin@ees.hokudai.ac.jp; 2 Course in Ecological Genetics, Division of Biosphere Science, Graduate School of Environmental Science, Hokkaido University, Kita-ku, Sapporo, Hokkaido 060-0810, Japan; mkk_hsmt@eis.hokudai.ac.jp; 3 Technical Solution Center First Group, J-OIL MILLS, Inc., Chuo-ku, Tokyo 104-0044, Japan; ryuji.hori@j-oil.com; 4 Laboratory of Environmental Microbiology, Division of Applied Bioscience, Graduate School of Agriculture, Hokkaido University, Kita-ku, Sapporo, Hokkaido 060-8589, Japan; takumin_ikuno@ybb.ne.jp; 5 Bioproduction Research Institute, Department of Life Science and Biotechnology, National Institute of Advanced Industrial Science and Technology (AIST), Toyohira-ku, Sapporo, Hokkaido 062-8517, Japan; 6 Laboratory of Environmental Molecular Biology, Section of Environmental Biology, Faculty of Environmental Earth Science, Hokkaido University, Kita-ku, Sapporo, Hokkaido 060-0810, Japan; hoku@ees.hokudai.ac.jp; 7 Department Food Science, Obihiro University Agriculture Veterinary Medicine, Inada-cho, Obihiro, Hokkaido 080-8555, Japan; yosori@obihiro.ac.jp; 8 ROM Co. Ltd., Togashi Bld., Chuo-ku, Sapporo, Hokkaido 060-0062, Japan; nagamine@rom-ef.co.jp; 9 Horonobe Research Institute for the Subsurface Environment, Northern Advancement Centre for Science and Technology, 5-3, Sakae-machi, Horonobe, Teshio-gun, Hokkaido 098-3221, Japan; satoru.shimizu@h-rise.jp (S.S.); akio.ueno@h-rise.jp (A.U.)

**Keywords:** long-chain polyunsaturated fatty acids, *pfa* genes, polyunsaturated fatty acid synthase (PUFA synthase), eicosapentaenoic acid (EPA), docosahexaenoic acid (DHA), arachidonic acid, very long chain polyunsaturated hydrocarbons

## Abstract

The nutritional and pharmaceutical values of long-chain polyunsaturated fatty acids (LC-PUFAs) such as arachidonic, eicosapentaenoic and docosahexaenoic acids have been well recognized. These LC-PUFAs are physiologically important compounds in bacteria and eukaryotes. Although little is known about the biosynthetic mechanisms and functions of LC-PUFAs in bacteria compared to those in higher organisms, a combination of genetic, bioinformatic, and molecular biological approaches to LC-PUFA-producing bacteria and some eukaryotes have revealed the notably diverse organization of the *pfa* genes encoding a polyunsaturated fatty acid synthase complex (PUFA synthase), the LC-PUFA biosynthetic processes, and tertiary structures of the domains of this enzyme. In bacteria, LC-PUFAs appear to take part in specific functions facilitating individual membrane proteins rather than in the adjustment of the physical fluidity of the whole cell membrane. Very long chain polyunsaturated hydrocarbons (LC-HCs) such as hentriacontanonaene are considered to be closely related to LC-PUFAs in their biosynthesis and function. The possible role of LC-HCs in strictly anaerobic bacteria under aerobic and anaerobic environments and the evolutionary relationships of anaerobic and aerobic bacteria carrying *pfa*-like genes are also discussed.

## 1. Introduction

The functions and structural nature of biological membranes are provided by the physical and chemical properties of their building blocks, lipids and proteins. The primary properties of lipids are largely specified by their constituting fatty acids; in turn, the properties of fatty acids are critically dependent on their chain length and degree of saturation. In bacterial cell membranes, saturated 16- and 18-carbon fatty acids are most common. Unsaturated fatty acids, usually containing one or two double bonds, occur as frequently as saturated fatty acids. However, some limited groups of bacteria have been demonstrated to produce distinct unsaturated fatty acids that have chain lengths longer than 20 carbons and contain at least four double bonds; examples include arachidonic acid (ARA, 20:4 *n*-6), eicosapentaenoic acid (EPA, 20:5 *n*-3), and docosahexaenoic acid (DHA, 22:6 *n*-3), which are collectively termed long-chain polyunsaturated fatty acids (LC-PUFAs) [1]. In nature there are two major modes of LC-PUFA biosynthesis. One is the mechanism by which LC-PUFAs are aerobically synthesized through the aerobic desaturation and elongation of saturated fatty acids that is typically employed in eukaryotes [2]. The other, the so-called polyketide mode that has been specifically found in bacteria, can anaerobically synthesize LC-PUFAs *de novo* [3]. The latter mode of reaction is catalyzed by an enzyme complex, PUFA synthase; the genes coding for the members of this complex are termed *pfa* genes [4].

The *pfa* genes that have been identified to be responsible for production of LC-PUFAs to date display a broad diversity of gene structure. To gain a comprehensive view of such a complex gene family, knowledge of the enzymatic domains that function in LC-PUFA biosynthesis is helpful. Here, we outline the reactions and enzymes in fatty acid biosynthesis because both biosynthesis pathways share many similar reactions [5]. Fatty acids, the constituents of lipids, and polyketides, which are classified as secondary metabolites, are primarily biosynthesized through the common type of carbon-chain building reaction wherein a carbon-carbon bond is formed by decarboxylative condensation utilizing a Claisen-type chemical reaction between acetyl-CoA as a starter unit and malonyl-CoA as an elongation unit. This reaction is carried out by three conserved functional components: an acyltransferase (AT), which loads the appropriate acyl group onto a reaction scaffold, a β-ketoacyl synthase (KS), which adds the loaded building block onto the growing acyl chain, and an acyl carrier protein (ACP), whose phosphopantetheine prosthetic group serves as the scaffold for the intermediate acyl chain during the entire elongation process. After condensation but prior to the next round of chain extension, in the fatty acid synthesis pathway the resulting β-keto group is processed via reduction and dehydration, which are performed by ketoreductase (KR), dehydratase (DH), and enoyl reductase (ER) enzymes, producing a β-hydroxyl, an α, β double bond, and a fully-reduced methylene, respectively. On the other hand, the polyketide biosynthesis pathways modify the growing polyketone-chain intermediates in various ways by re-arranging the order and combinations of these reductive enzymatic components to produce diverse final products including antibiotics, toxins, pigments, and infochemicals [6]. In this review, we first describe the structure and domain organization of the *pfa* genes, and then discuss the process of LC-PUFA biosynthesis in bacteria.

Since the melting temperatures of LC-PUFAs are much lower than those of saturated and monounsaturated fatty acids, appropriate membrane fluidity at low temperatures can be attained by membrane phospholipids containing LC-PUFAs. Therefore, LC-PUFAs, particularly EPA and DHA, have been believed to be efficient modulators for adjusting membrane fluidity. In fact, LC-PUFAs are detected exclusively in bacteria that inhabit cold marine environments such as the Polar Regions, deep seawater, and within sea fishes in general. These bacteria produce much higher levels of LC-PUFA when grown at lower temperatures. In addition, it has been observed that DHA-producing bacteria are more abundant in deeper seawater (a lower temperature environment) than are EPA-producing bacteria [7]. This trend has been considered to be explained by the fact that the melting temperature of DHA is lower than that of EPA. Therefore, the view that LC-PUFAs in the cell membrane are important factors for cold adapted bacteria has been commonly shared by researchers. However, such a classical concept for the function of LC-PUFAs has not been necessarily confirmed functionally in LC-PUFA-producing bacteria or eukaryotic microorganisms.

Recent progress in genetic engineering has allowed these techniques to be applied to such bacteria to elucidate the physiological roles of LC-PUFAs and has provided new findings regarding the function of LC-PUFAs, particularly of EPA, in bacteria. In some EPA-producing psychrophilic and piezophilic bacteria, EPA was found not to be involved in their adaption to cold-temperature and high-pressure environments; rather, the presence of EPA constrains the membrane fluidity. Another unexpected new function of EPA and DHA is that these LC-PUFAs have been shown to be involved in the resistance of bacteria against extracellular oxidants such as H_2_O_2_. As LC-PUFAs have many bisallylic hydrogen atoms, which are quite active toward reactive oxygen species (ROS) and free radicals [8,9], it was conceivable that these compounds are apt to be easily oxidized by oxidants (or ROS). However, LC-PUFAs located in cell membrane are not oxidized by (or are rather stable against) exogenously added ROS [10]. The antioxidative function of LC-PUFAs is difficult to be conceptualized and its molecular mechanism is to be solved. On the other hand, EPA is responsible for cell division only at low temperatures in some EPA-producing marine bacteria suggesting that its presence is not entirely a consequence of its membrane fluidity adjustment. These findings were provided by investigations employing various EPA-producing marine bacteria and their EPA-deficient mutants in addition to *Escherichia coli* (*E. coli*) recombinants transformed by the *pfa* genes responsible for EPA or DHA biosynthesis. Recently, much attention has further been paid to the relationships between LC-PUFAs and individual membrane functions such as membrane transport and the efflux activities of various compounds. We describe here the functions of LC-PUFAs with an emphasis on their involvement in the efflux of antibiotics in *E. coli* recombinants capable of producing EPA.

Hentriacontanonaene (C_31_H_46_; C31:9), a very long-chain polyunsaturated hydrocarbon (LC-HC), was originally discovered in a psychrophilic bacterium isolated from Antarctic sea ice cores [11]. It was subsequently demonstrated that all C31:9-producing bacteria are facultative anaerobes and simultaneously produce EPA or DHA [12]. C31:9 is predicted to be synthesized by the condensation of two 4,7,10,13-hexadecatetraenoic acid (16:4 *n*-3) molecules and subsequent reactions, which are catalyzed by OleA, B, C, and D [13]. A medium chain polyunsaturated fatty acid, 16:4 *n*-3 is a product of the PUFA synthase PfaA-E [12]. Since the amount of C31:9 significantly increases in cells grown at decreased temperatures, this hydrocarbon has been suggested to play a role in cold adaptation [13]. On the basis of the fact that strictly anaerobic bacteria such as *Geobacter bemidjiensis* (*G.* *bemidjiensis*) Bem^T^ contain a cluster of *pfa* and *ole* genes found in tandem, we finally discuss the physiological significance of the Pfa and Ole protein products in strict anaerobes and the evolutionary implications of the phylogenetic distribution of the *pfa* and/or *ole* genes and their homologs in bacteria.

## 2. Organization and Function of the *pfa* Genes

### 2.1. Cluster Structure and Domain Organization of the pfa Genes

Fatty acid or polyketide *de novo* biosynthesis is achieved by fatty acid (FAS) or polyketide (PKS) synthases, respectively. According to the architecture and mode of employment of the enzymatic components, FASs and PKSs are generally classified into three types. A type I synthase is a multifunctional megasynthase consisting of multiple domains, each of which functions as an enzymatic component linked together in a single polypeptide. In contrast, a type II synthase is a discrete and monofunctional enzyme that functions as a dissociable complex. Additionally, a multifunctional enzyme of the chalcone synthase group is classified as a type III PKS and can synthesize a final product without the support of an ACP [14].

**Figure 1:**
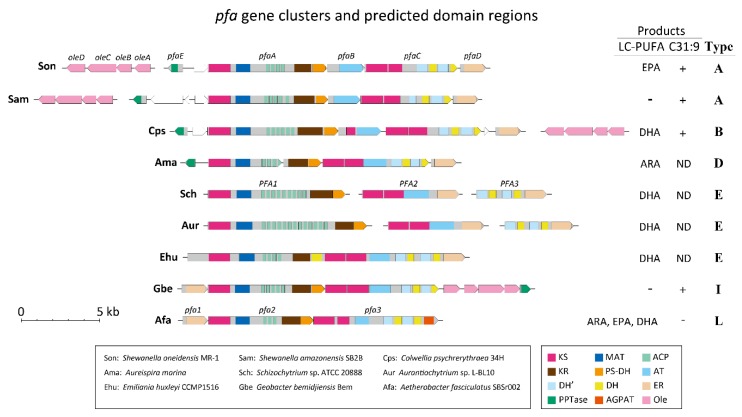
Examples of *pfa* and *ole* gene clusters. Gene clusters of *pfa* gene families and *ole* genes from nine microbial genomes are shown. Horizontal lines indicate genome sequences. Lines split into several parts denote that separated genome regions are located in different loci or have not yet been mapped. Gray colored boxes show *pfa* gene coding regions with their enzymatic domains indicated by colored boxes. White pentagonal boxes represent genes unrelated to *pfa* genes. Pink colored pentagonal boxes are *ole* genes. The acute angles of the pentagonal boxes indicate the direction of transcription. Gene names if assigned are listed on the boxes. The domain regions were located by NCBI BLASTP searches; these include β-ketoacyl synthase (KS), malonyl-CoA acyltransferase (MAT), acyl carrier protein (ACP), ketoreductase (KR), polyketide synthase dehydratase (PS-DH), acyltransferase (AT), dehydratase (DH), enoyl reductase (ER), phosphopantetheine transferase (PPTase), 1-acylglycerol-3-phosphate *O*-acyltransferase (AGPAT), and *oleA*–*D*. DH’domains were also identified by NCBI BLASTP searches with amino acid sequences that span regions 1096–1305 and 1498–1755 of PfaC in *Photobacterium profundum* SS9 [15]. In the table, “+” denotes that C31:9 was detected, and “−” denotes that products were not detected. ND means that the existence of C31:9 has not been determined. *pfa* gene clusters are classified into Types according to Shulse and Allen [16].

LC-PUFAs found in microorganisms are also biosynthesized *de novo* by the FAS/PKS like enzyme system, namely PUFA synthase [3]. Since 2000, many *pfa* gene clusters have been identified, primarily within marine microorganisms that produce LC-PUFAs, and it has been found that the gene organization, order of genes, and composition of enzymatic domains are highly conserved [1]. Figure 1 shows representative gene structures and enzymatic domains of the *pfa* gene clusters responsible for the production of LC-PUFAs. The multidomain organization of PUFA synthases resembles that of FAS/PKS and their multigene system and the gene clustering is reminiscent of the similar feature typical of type II bacterial FAS systems. This complex organization might reflect the evolutionary history of the *pfa* gene clusters and/or an adaptive or structural significance of PUFA synthases.

The conserved arrangements of the *pfa* genes are observed not only in LC-PUFA producing bacteria but also in ecologically and phylogenetically widespread bacterial species. Shulse and Allen [16] identified *pfa* gene homologs from the various bacterial genomes of 86 species belonging to 10 phyla and classified them into 20 types (A–T) based on the combination of domain arrangements and the final products, if known. Although the fatty acyl metabolites, designated as “secondary lipids”, synthesized by these *pfa*-like gene products are largely unknown, the gene types show a significant correlation with diverse ecological and physiological properties of the microorganisms possessing these genes. Therefore, the authors point out that a deeper understanding of secondary lipid biosynthesis pathways expands our insight into the ecological adaptation and evolution of microorganisms.

Since *pfa* gene structures vary considerably, we herein adopted the *pfa* gene cluster of *Shewanella oneidensis* (*S. oneidensis*) MR-1 as a typical structure (Figure 1). The genes responsible for EPA production in *S. oneidensis*, a mesophilic bacterium, consist of five open reading frames (ORFs) denoting the *pfaA*–*E* genes, which form a gene cluster in the genome [17]. *pfaA* is a multi-functional gene that includes five domains: KS, malonyl-CoA acyltransferase (MAT), four repeats of ACP, KR, and PKS-like DH. *pfaB* is mono-functional, possessing one AT domain. *pfaC* includes two KS and four FabA-like DH domains (see below). The *pfaD* gene has only a single domain, ER. The last *pfaE* gene, which encodes phosphopantetheine transferase (PPTase), is located upstream of the *pfaA* gene and is oriented in the opposite direction. Based on this gene organization, this *pfa* gene cluster is classified into Type A [16].

In almost all eukaryotes, most unsaturated fatty acids including LC-PUFAs are commonly produced by a combination of fatty acid elongation and oxygen-dependent desaturation. However, some marine algae have the potential to synthesize LC-PUFAs using *pfa* genes whose organization is similar to those of bacteria (Figure 1). *Schizochytrium* sp. and *Aurantiochytrium* sp. have a *pfa* organization consisting of *PFA1* (equivalent to *pfaA*), *PFA2* (equivalent to *pfaB* + the anterior region of *pfaC* + *pfaD*), and *PFA3* (the posterior region of *pfaC* + *pfaD*). Notably, a haptophyte *Emiliania huxleyi*, which produces mainly DHA, exhibits a quite unique *pfa* gene organization consisting of only one ORF with a structure the same as that of the combined *PFA1*, *PFA2*, and *PFA3* of *Schizochytrium* sp. and *Aurantiochytrium* sp., in this order. These gene clusters are classified into Type E [16].

Recently, two new *pfa* genes have been experimentally verified to be responsible for LC-PUFA biosynthesis. *Aureispira marina* (*A. marina*), which is classified into the phylum Bacteroides and was isolated from a marine sponge and an alga that inhabit the coastal areas of Thailand, produces 30%–40% of its total fatty acids (TFAs) as ARA [18]. The *pfa* gene homologs that were isolated from the bacterial genome direct ARA biosynthesis when they are introduced into *E. coli* [19]. Although the genomic structure of the genes highly resembles the Type A *pfa* gene cluster with respect to domain arrangement, the region containing the KR domain is separate from the *pfaA* gene, and the AT domain region is fused to the *pfaC* gene (Figure 1). Based on this domain organization and the production of ARA, the *pfa* gene cluster in *A. marina* can be grouped into Type D. This category also includes the gene cluster found in a marine Bacteroidetes species, *Psychroflexus torquis* [16], which is a psychrophilic species isolated from Antarctica ice samples [20]. Despite remarkable difference in their habitats, the finding that *A. marina* and *Psychroflexus torquis* share conserved *pfa* genes and ARA production might be of considerable relevance toward understanding the physiological function of LC-PUFAs.

Although the *pfa* genes have been almost exclusively found from marine bacteria, another example of a *pfa* gene cluster has been identified in terrestrial myxobacteria of the genus *Aetherobacter* [21]. Four species of *Aetherobacter* produce several types of LC-PUFA including ARA, EPA, DHA, and C18 PUFAs. The *pfa* gene clusters found in these bacteria consist of three ORFs (*pfa1–3*) and show several structural differences when compared with a typical Type A cluster (Figure 1). The *pfaD* gene homolog containing an ER domain is located upstream of the myxobacterial gene cluster, and the *pfaB* gene segment is missing from the cluster of *Aetherobacter* spp.; instead, the AT domain present in the *pfaB* gene is relocated in the middle of the *pfa3* gene. In addition, the *pfaE* gene homolog that encodes a PPTase is found at a distinct locus separated from the *pfa* gene cluster, as was seen in *Moritella marina* (*M. marina*) MP-1 [22]. The most characteristic feature of the myxobacterial *pfa* genes is that the *pfa3* gene corresponding to the *pfaC* gene possesses a domain coding for 1-acylglycerol-3-phosphate *O*-acyltransferase (AGPAT) at its carboxyl terminal. Judging from the gene organizational features, the *pfa* genes of *Aetherobacter* spp. can be classified into Type L. It is unclear whether the myxobacterial PUFA synthases can produce all of the types of LC-PUFAs found in these bacteria or whether they exclusively produce only certain LC-PUFAs, which are then converted into the remaining LC-PUFAs by other enzyme activities. *Sorangium cellulosum* (*S. cellulosum*), a closely related species to *Aetherobacter*, produces only linoleic acid (LA) as a PUFA, but not any other LC-PUFAs [23]. Although both bacteria share the highly conserved *pfa* genes, the *pfa3* gene of *S. cellulosum* lacks the AT domain sequence [21]. This discrepancy might explain the inability to synthesize EPA and DHA in *S. cellulosum*.

### 2.2. Biosynthetic Process of LC-PUFAs in Bacteria

#### 2.2.1. Initiation

The LC-PUFA biosynthesis reaction is initiated by activating the ACPs of PfaA from their apo- to their active holo-form. This activity is catalyzed by PPTase (PfaE), which transfers a 4′-phosphopantetheine prosthetic group from CoA to a serine residue of ACP [24]. PfaE is considered to utilize the ACP repeats of PfaA specifically to activate ACP. However, the heterologous production of EPA was observed in *E. coli* transformed in combination with the *pfaA*–*D* gene from *Shewanella* *pneumatophori* (*S*. *pneumatophori*) as well as with the *E. coli entD* gene encoding an Sfp-type PPTase responsible for the biosynthesis of siderophore [25], indicating that the activation of PfaE can be replaced partially by that of other types of PPTases.

#### 2.2.2. Extension of Acyl Chains (Elongation, Double Bond Formation, and Final Product Determination)

The activated ACP domains of PfaA, hereafter designated as PfaA(ACP), accept acetyl and malonyl groups from acetyl- and malonyl-CoA and each reaction is respectively catalyzed by AT and MAT. These two molecules on the PfaA(ACP) are condensed with the release of one molecule of CO_2_ by a Claisen condensation reaction catalyzed by KS. Subsequently, the β-oxo group is reduced by KR and the β-OH group is formed. The latter undergoes a dehydration reaction catalyzed by DH to form a Δ^2^-*trans* double bond; this double bond then is reduced by ER to form a saturated C–C bond or isomerized to Δ^2^-*cis* and Δ^3^-*cis* isomers by DH. The thus-formed *cis* double bonds are conserved throughout LC-PUFA synthesis. The Type A PUFA synthase system has a PKS-DH on PfaA and a dual FabA DH in the PfaC; however, their role-sharing has not been clarified. The cycle of condensation by KS and modification by KR, DH, and ER extend the fatty acyl chains in two carbon increments. These sequential reactions in LC-PUFA biosynthesis are speculated based on the functional domain structures of the *pfa* genes. However, confirmation of the individual enzymatic reactions of the gene products have not yet been fully determined.

Type A, B, and D *pfa* gene clusters are involved in the synthesis of EPA, DHA, and ARA, respectively (Figure 1). Although the mechanism determining the final LC-PUFA product by the PUFA synthase is not yet fully understood, the KS domain, which exists only in the PfaB of the Type B *pfa* cluster, is known to be responsible for the synthesis of DHA [26]. Furthermore, the characteristic domain order in the Type D cluster, in comparison with that in Type A, is that the position of the AT domain moves between the carboxyl-terminal KS domain and the DH’ domain (Figure 1); thus, this feature might be involved in determining the type of final LC-PUFA produced.

#### 2.2.3. Chain-Termination of LC-PUFA Biosynthesis and Their Integration into Lipids

The LC-PUFA synthesis process is accomplished when the final products are incorporated into lipids. Newly synthesized LC-PUFAs are transferred to 1-acyl-3-gycerophosphate by AGPAT, forming phosphatidic acid, and the final acceptor molecules of these constitute phospholipids in bacteria or phospholipids and triacylglycerols in eukaryotes.

**Figure 2:**
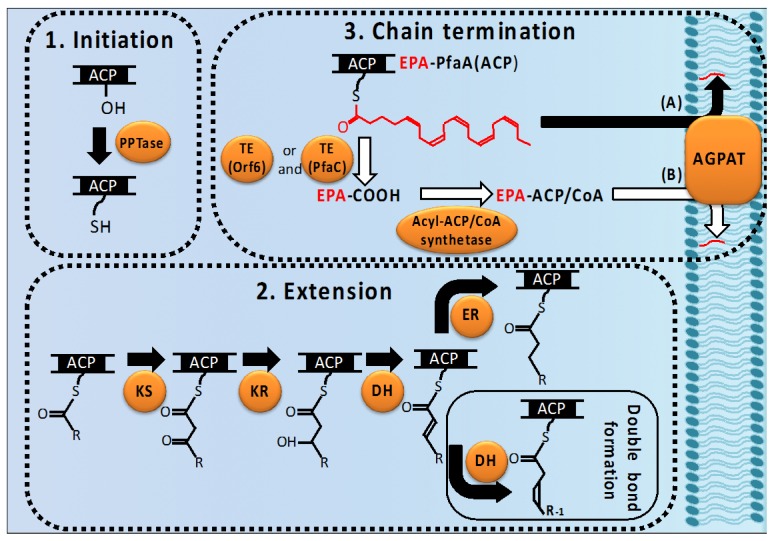
Possible processes for LC-PUFA biosynthesis via PUFA synthase and their incorporation into phospholipids by membrane-bound 1-acyl-3-phosphatidic acid acyltransferase (PlsC). Fatty acid LC-PUFA biosynthesis is initiated by activation of the acyl carrier protein (ACP) domains of the Pfa enzyme PUFA synthase via 4′-phosphopantetheine transferase (PPTase) (1. Initiation). The extension of an acyl chain is carried out by the combined β-ketoacyl synthase (KS), ketoreductase (KR), and bifunctional dehydratase (DH) activities (2. Extension). The growing fatty acids (blue wavy line) with a Δ^2^-*trans* double bond are reduced to form saturated fatty acids catalyzed by enoyl reductase (ER), and those with a Δ^2^-*cis* or Δ^3^-*cis* double bond are isomerized positionally and geometrically to form unsaturated fatty acids catalyzed by bifunctional PKS or FabA DH. In the termination step, the matured LC-PUFA molecule, represented by EPA in this figure (red wavy line), which is accommodated in the Pfa protein is either directly used as substrate of the PlsC to synthesize phospholipids (route A; black arrow) or released to free acid by thioesterase (TE) encoded by *orf6*. In the latter step, free LC-PUFAs are converted to CoA/ACP derivatives by acyl-CoA/ACP synthetase, which are then used for phospholipid synthesis by PlsC (route B; white arrow) (3. Termination).

*Shewanella livingstonensis* (*S. livingstonensis*) Ac10, which produces EPA, possesses five types of genes homologous to *E. coli* *plsC* (AGPAT). Notably, the bacterial mutant for one of these (*plsC1*) synthesizes significantly reduced levels of EPA [27], indicating that EPA is transferred following completion by *PlsC1*. Thus, two schemes for the chain-termination and transfer route of LC-PUFAs are generally considered in bacteria (Figure 2). One is the pathway wherein the LC-PUFAs synthesized on PfaA(ACP) are directly utilized as a substrate of transacylation to generate phosphatidic acid. In this case the transfer of acyl groups catalyzed by AGPAT is regarded as a chain-terminating step in the synthesis of LC-PUFA. AGPAT in LC-PUFA producing bacteria might use acyl-ACP and/or acyl-CoA for its substrate because of its high similarity to the *E. coli* *PlsC*, which utilizes either compound as a substrate. This raises the possibility that AGPAT recognizes LC-PUFA-PfaA(ACP) as a substrate as well.

The second possibility is that thioesterase (TE) works as a chain-terminator in the biosynthesis cycle of LC-PUFA. Type I FAS/PKS multidomain enzymes generally have TE domains to cleave their final products from ACP domains. In PUFA synthase systems, Rodriguez-Guilbe *et al.* [28] found that bacteria possessing *pfa* gene clusters belonging to Type A or B share highly conserved TE genes; in particular, *Photobacterium profundum* (*P. profundum*) SS9 (Type A) and *M. marina* MP-1 (Type B) genomes possess TE genes located immediately upstream of their *pfaA* genes. They also showed that the purified TE proteins exhibit a TE activity specific for long-chain fatty acyl-CoAs such as palmitoyl- or eicosapentaenoyl-CoA.

The action of TE as a chain-terminator releases the LC-PUFA molecules synthesized as acyl-ACP derivatives on the PfaA(ACP). The released free LC-PUFAs must be then activated to form LC-PUFA-CoA by acyl-CoA synthetase or LC-PUFA-ACP by acyl-ACP synthetase, which utilizes an ACP as an acceptor for free LC-PUFA. Acyl-CoA synthetase genes are found in both Type A and B bacterial genomes and acyl-ACP synthetase genes are present in Type A, suggesting that in these bacteria the LC-PUFAs released by TE are loaded onto either ACPs or CoAs by these synthetases and are then transferred into phospholipids by AGPAT. If this scenario is correct, TE must be an important enzyme for the PUFA synthase system. Therefore, we propose here to use *orf6* to designate the TE gene, because the corresponding TE gene of *P. profundum* SS9 has been called as *orf6* [28].

In addition, EPA can be synthesized in *E. coli* recombinants carrying only the *pfaA–E* genes from *S. pneumatophori* but without *orf6* [29], indicating that the *orf6* gene is not essential for LC-PUFA biosynthesis. *E. coli* exhibits minimal acyl-ACP TE activity; therefore, these recombinants would not have been able to release LC-PUFAs from PfaA(ACP) via endogenous bacterial enzymes, implying that the heterologously expressed PUFA synthase exhibits LC-PUFA releasing activity, acting as a TE. This postulated third mechanism is suggested by several studies. First, an *E. coli* recombinant expressing the DNA sequence region covering the DH domain of the *P. profundum* SS9 *pfaC* gene was shown to be capable of producing up to a 5-fold increase in TFAs over the negative control, although the dehydration reaction had not previously been identified as a limiting step in bacterial fatty acid biosynthesis [30] and overexpression of the native dehydratase from *E. coli*, FabA, did not increase the production of fatty acids [31]. Therefore, it was proposed that this DH domain also catalyzes the reaction of thioester hydrolysis (TE activity). In addition, the corresponding DH domain of the PfaC family is composed of four contiguous hotdog fold domains, described in detail in Section 2.3, characteristic of dehydratases and thioesterases and this family of structural domains has elsewhere been implicated in both DH and TE activity [32].

Furthermore, since the intrinsic TE activity and the distinct *orf6* function can be compatible with each other, some LC-PUFA producing bacteria are likely to contain both of these chain-termination modes simultaneously.

### 2.3. Tertiary Structures of the Domains

The determination of three-dimensional enzyme structure helps to understand enzyme kinetics and reaction mechanisms. Although the conformation of PUFA synthases has not yet been solved, Baerga-Ortiz and his colleagues have recently reported the tertiary structures of two domains, DH and ACP, from the PUFA synthase [15,33]. Intensive sequence analysis in the C-terminal region of the *pfaC* gene revealed two hidden pseudo-DH domains, which show relatively high sequence similarity to each other but lack a conserved active site His residue. Each pseudo-DH domain is located immediately upstream of two DH domains previously known to be related to a FabA DH domain. Furthermore, an estimated three-dimensional modeling of these DH and pseudo-DH domains showed that not only the DH domains but also two pseudo-DH domains were predicted to form so-called “hotdog” folds. This conserved structural motif, which is recognized in many dehydratases such as bacterial FabA and FabZ and thioesterases, possesses a long central α-helix “sausage” that nestles in a seven-stranded antiparallel β-sheet “bun” [32]. Although it has not yet been demonstrated experimentally how these four tandemly arranged hotdog folds are formed into the functional assembly, if these domains comprise the two pseudodimeric DHs in the LC-PUFA biosynthesis process, each postulated DH dimer might catalyze different reactions, *i.e.*, *trans*-2,2-*cis* isomerization and *trans*-2,3-*cis* isomerization, respectively, required to introduce double bonds.

Normally, each FAS/PKS ketosynthase module contains a single ACP domain whereas PUFA synthases are characterized by the presence of multiple ACP domains, ranging from two to over 10. An increased number of ACP domains in PUFA synthases appear to contribute to an additive effect of LC-PUFA yield [34]. Furthermore, in the PKS for curacin biosynthesis in cyanobacteria evaluation of the contribution of each triplet ACP domains revealed that individual ACP domains act in parallel and are functionally equivalent [35]. To understand the role of multiple ACP domains, Trujillo and co-workers reconstructed three-dimensional models of the five ACP domains tandemly arranged in the PUFA synthase of *P. profundum* [33]. In this study, small-angle X-ray scattering (SAXS) analysis was used to predict the solution structure of the tandem ACP [36]. The most plausible model calculated from the SAXS data shows a monomer form and an elongated beads-on-a-string structure. In addition, the SAXS analysis revealed that the tandem ACP could adopt several conformations in solution, suggesting that the structural flexibility of the tandem ACP would have additive and parallel effects on LC-PUFA production. The crystallization of modular multidomain proteins with flexible linkers is often problematic owing to their higher flexibility in solution, making it difficult to analyze their structure by X-ray crystallography. Therefore, the SAXS technique, in which sample crystallization is unnecessary, and sophisticated modeling programs that utilize SAXS data are likely to be increasingly applied to the structural determination of flexible multidomain proteins including PUFA synthases [37].

## 3. Functions of LC-PUFAs in Bacteria and Their Practical Use

### 3.1. Involvement of LC-PUFAs in Cold Adaptation

Most LC-PUFA-producing bacteria have been isolated from marine sources such as seawater, fish, and sediments and have been commonly psychrophilic (psychrotrophic) and/or piezophilic in nature. Therefore, the functions of LC-PUFA in such bacteria have been discussed in relation to their adaptation to low-temperature and high-pressure environments. The relevant bacteria spontaneously increase their content of LC-PUFA like EPA or DHA when grown under lower temperatures or higher pressures [38]. Therefore, the function of LC-PUFAs in these bacteria has been regarded as the adjustment of cell membrane fluidity under these conditions [39] although this classical concept has yet to be experimentally confirmed in either bacterial or eukaryotic LC-PUFA-producing microorganisms. Recently it was evidenced that EPA is not essential in some EPA-producing bacteria and that the loss of EPA appears to be readily compensated by increased levels of monounsaturated fatty acids (MUFAs) or branched chain fatty acids (BCFAs) (see below). For example, the psychrotolerant piezophilic deep-sea bacterium *P. profundum* SS9 produces EPA (8% of TFAs) and the content of EPA increases when grown under low temperatures and high pressures [40]. However, growth inhibition of the *P. profundum* SS9 mutant strain EA10, which is defective in EPA synthesis, is completely compensated *in vivo* by the increased content of MUFAs. In contrast, the *P. profundum* SS9 mutant strain EA3, which is deficient in the production of MUFAs, becomes sensitive to low temperature and high pressure and this sensitivity is relieved by exogenously added MUFA but not by EPA [40]. In addition, the EPA-producing psychrotrophic bacterium *Shewanella marinintestina* (*S. marinintestina*) IK-1, isolated from the intestine of a squid, has an EPA content representing approximately 15% of TFAs at 4 °C but which is almost negligible at 27 °C. We observed that mutant IK-1Δ8 cells, deficient in EPA production, grow as well as the original IK-1 cells even at 4 °C [41], indicating that EPA does not play an important role in the growth of this species at lower temperature. Together, these studies using EPA-deficient mutants indicate that EPA is not primarily responsible for adjusting the membrane fluidity at low temperature or high pressure conditions.

EPA is absolutely required for the growth of bacteria at low temperatures and high pressures in some *Shewanella* species. *S. livingstonensis* Ac10, an isolate from seawater of the Antarctic sea, is a psychrotrophic bacterium capable of producing EPA [42]. The EPA content of the membrane phospholipids of *S. livingstonensis* Ac10 grown at 4 °C is approximately 5% of TFAs whereas the EPA content is almost negligible at 18 °C. Ac10 EPA-deficient mutants grow as well as the wild-type strain at 18 °C. However, mutant growth is significantly inhibited at 4 °C and this growth inhibition is recovered only when EPA-containing phospholipids are added into growth medium, suggesting that EPA is important for *S. livingstonensis* Ac10 to grow at lower temperature [42]. *Shewanella violacea* (*S. violacea*) strain DSS12 is a deep-sea bacterium isolated from the Ryukyu Trench. It exhibits moderate piezophily with optimal growth at 30 MPa and 8 °C but it can also grow at 0.1 MPa. *S. violacea* DSS12 cells contain a substantial amount of EPA (15% at 8 °C) in their membrane [43]. Usui *et al.* [44] measured the dynamic properties of these cells using a new system that enables the measurement of fluorescence anisotropy under high pressure and demonstrated that in this bacterium EPA appears to structurally maintain cell membrane rigidity by affecting membrane hydration over a wide range of pressure conditions. EPA also plays a role in *S. violacea* DSS12 cell division under high pressure because the cell shape of an EPA-deficient DSS12 mutant becomes filamentous at 30 MPa. In comparison, *Shewanella piezotolerans* (*S. piezotolerans*) WP3, which was isolated from a sediment sample of the western Pacific Ocean, is a psychrotolerant and piezotolerant bacterium that grows optimally at 15–20 °C under pressures of 0.1–20 MPa [45]. This bacterium also requires EPA for its growth at low temperatures and high pressures. Its EPA content is approximately 14% and 6% of TFAs at 4 °C and at 20 °C, respectively, and much higher levels of EPA are detected in cells grown under pressures of 20 MPa than under 0.1 MPa. In this bacterium, however, there is a much greater requirement for branched iso-13:0 and iso-15:0 fatty acids (BCFAs), whose combined levels are approximately 50% and 22% of the total at 4 °C and at 20 °C, respectively, than for EPA [46]. This differs from *P. profundum* SS9, which absolutely requires MUFAs at low temperatures. Furthermore, the growth retardation of an EPA-deficient mutant of *S. piezotolerans* (WP3^ΔEPA^) at 4 °C is partly compensated by increased levels of BCFAs [46]. Accordingly, the exact function of EPA is still unknown in this bacterium. However, EPA might affect the activity of the branched-chain amino acid ABC transporter involved in BCFA biosynthesis in *S. piezotolerans* WP3, because this transport system is upregulated only at low temperatures [46]. Therefore, although these examples indicate that EPA is required for the growth of various *Shewanella* species in low temperature and/or high pressure conditions, its role is likely specific to individual membrane functions but not to overall membrane fluidity (see below for details).

Certain *Schizochytrium* and related heterotrophic eukaryotic microorganisms are known to produce and accumulate notable levels of LC-PUFAs (up to 50% of TFAs) through PUFA synthases encoded by the bacterial *pfa*-like genes (Figure 1). These organisms have a capacity to produce LC-PUFAs at 25 °C or much higher temperatures. To examine the role of LC-PUFAs, Lippmeier *et al.* [47] generated DHA-deficient mutants of *Schizochytrium* sp. (ATCC 20888) by disrupting one of the three *PFA* genes. The resulting DHA-deficient mutants became auxotrophic to LC-PUFA and required supplementation of unsaturated fatty acids such as ARA, EPA, and DHA for its normal growth [47]. As *Schizochytrium* sp. ATCC 20888 lacks a Δ^12^ fatty acid desaturase activity, LC-PUFAs synthesized through the *pfa*-like genes are therefore absolutely necessary in this organism to maintain the membrane structure and functions even at moderate temperature, which is in complete contrast with bacteria cells wherein LC-PUFAs are required only under low temperature and high pressure conditions. However, the function of LC-PUFAs in *Schizochytrium* sp. ATCC 20888 and other eukaryotic microorganisms possessing the *pfa*-like genes has not been certified.

### 3.2. Antioxidative Functions of LC-PUFAs

Because ROS such as H_2_O_2_ are freely membrane-permeable compounds, bacteria cope with ROS attacks by using catalase or other types of ROS scavenging enzymes. Since the cell membrane of most bacteria is composed of saturated and monounsaturated fatty acids [48], these fatty acids in the membrane are not regarded as the primary targets of oxidative stress caused by exogenous ROS agents. In contrast, PUFAs including LC-PUFAs are readily susceptible to oxygen or ROS; thus, bacterial membranes with LC-PUFAs are considered much more susceptible to oxidants. Notably, however, bacteria with LC-PUFAs are unexpectedly more resistant to ROS than those without LC-PUFAs [10,49].

The antioxidative function of LC-PUFAs in bacteria was reported for the first time by Nishida *et al.* [10]. *E. coli* recombinants genetically engineered to produce EPA (designated EPA+) become more resistant to exogenously supplied ROS such as H_2_O_2_ and *tert*-butyl hydroperoxide (*t*-BHP) compared to the parent strain that produces no EPA (designated EPA−). The antioxidative function of EPA was confirmed using natively EPA-producing *S. marinintestina* IK-1 and its EPA-less mutant (IK-1Δ8) [50,51,52]. Recently, Tilay and Annapure [53] successfully isolated EPA-producing and ARA-producing bacteria from seawater samples by evaluating resistance to H_2_O_2_. This report supports the concept of an antioxidative function of not only *n*-3 LC-PUFAs but also *n*-6 LC-PUFAs such as ARA as well. Since the resistance of *E. coli* DH5α recombinants with EPA to H_2_O_2_ depends on their cellular levels of EPA [10], relatively high levels of EPA in membrane phospholipids might be required for the antioxidative activity in bacteria. Consistent with this hypothesis, catalase-deficient *E. coli* UM2 mutant cells transformed with the *pfaA–E* genes can produce EPA at levels of 7%–8% of the TFAs and become more resistant than the reference strain without EPA [49].

As a possible mechanism responsible for the antioxidative function of EPA and other LC-PUFAs, a cell membrane-shielding effect of LC-PUFAs has been proposed [50]. In this mechanism, a more hydrophobic interface (region) of the alkyl chain is suggested to be formed between the phospholipid bilayers when cell membrane phospholipids are acylated in combination with a PUFA and a medium-chain saturated or monounsaturated fatty acid [54]. The resulting hydrophobicity renders it difficult to pass extracellular hydrophilic compounds through the membrane. Simultaneously, the hydrophobicity allows the ready passage of hydrophobic compounds. Thus, using this relationship we can evaluate the membrane-shielding effect by measuring its hydrophobicity. To examine this effect, we obtained several *E. coli* DH5α recombinant cell lines that exhibit different levels of EPA by introducing different combinations of the *pfa* genes and found that the cell line with the highest EPA level exhibited higher cell hydrophobicity than that with the lowest EPA level [55]. The antioxidative function of EPA (and of other LC-PUFAs) is predicted to be based on their common structural and hydrophobic characteristic in the bacterial cell membranes.

### 3.3. Involvement of LC-PUFAs in Specific Membrane Functions

*S. livingstonensis* Ac10 requires EPA for proper growth at lower temperatures as described above. Notably, its EPA-defective mutants display almost the same levels of cell membrane fluidity as that seen in normal cells [42]. However, the mutant cells at lower temperature show an abnormally elongated cell morphology during cell division and a significantly lower growth rate [42]. Tracer experiments in this bacterium at 4 °C using fluorescence-labeled phospholipids containing EPA revealed that the fluorescent signals localized between two nucleoids at the cell center during cell division, whereas no particular localization was observed for labeled phospholipids containing oleic acid [56]. This localization of EPA-containing phospholipids (EPA-PLs) suggests that these are required for cell membrane curvature at the occlusion site during cell division or, alternatively, that they interact with membrane proteins at the site of cell division. The latter possibility might be supported by the result that the outer membrane protein Omp74, which is induced at lower temperatures in *S. livingstonensis* Ac10, requires liposomes with EPA-PLs for the acceleration of its proper folding and formation of its β-sheet structure *in vitro* [57]. These findings suggest that at lower temperature environments EPA-PLs act as a chemical chaperon for membrane proteins and contribute to the formation of a microdomain to facilitate cell division.

**Figure 3:**
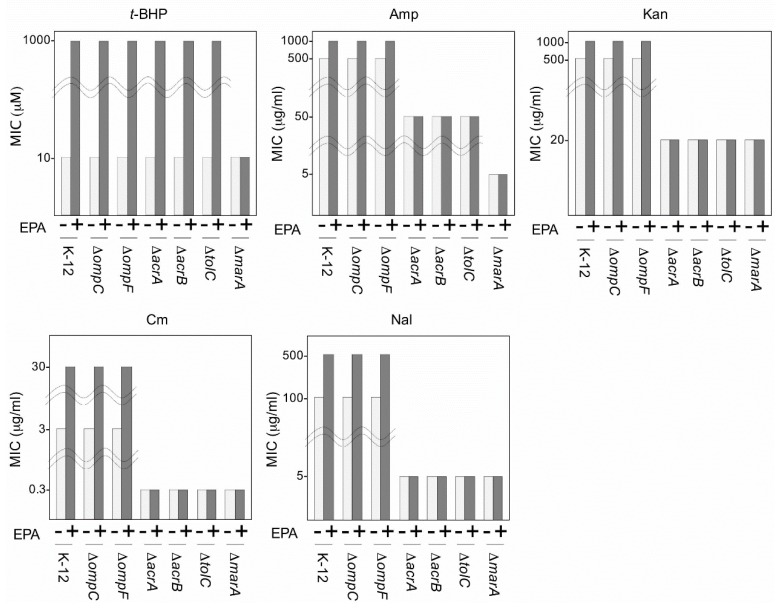
Effect of *tert*-butyl hydroperoxide (*t*-BHP) and various antibiotics on the growth of *Echerichia coli* (*E. coli*) K-12 and its mutants transformed with the clustered *pfa* genes for EPA biosynthesis. To perform the growth inhibition tests, 96-well microtiter plates were used as described previously [51]. The plates were incubated for 4 days at 20 °C. EPA+ and EPA− exhibited EPA production and lack of production, respectively. Amp, ampicillin; Kan, kanamycin; Cm, chloramphenicol; Nal, nalidixic acid. MIC, minimum inhibitory concentration. *E. coli* K-12 and its mutants used in this study were purchased from the Coli Genetic Stock Center, Yale University.

Both *E. coli* DH5α that heterologously express EPA and *S. marinintestina* IK-1 that intrinsically produce EPA show increased resistance against antibiotics [51,55]. This multidrug resistance mechanism might be explained by two possible EPA effects on the cell membrane: One such possibility is the cell membrane-shielding effect of EPA as described above. The other is an interaction of EPA with membrane proteins involved in drug resistance. To distinguish between these, we investigated the influence of EPA on mutants in proteins that affect drug permeation and efflux, and examined the cell membrane-shielding effect of EPA on these mutants [41]. *E. coli* expresses the Omp system, which is involved in membrane permeation and includes the outer membrane porin proteins, OmpC and OmpF [58]. *E. coli* also contain the AcrAB-TolC multidrug efflux system [59], which consists of AcrA, AcrB, and TolC, and utilizes MarA as a regulator for the *acrA* and *acrB* genes. We generated two groups of bacterial lines; the first group (EPA+) included EPA-producing *E. coli* K12 recombinants and its mutants deficient in *ompC*, *ompF*, *acrA*, *acrB*, *tolC*, or *marA*, which were transformed with the *pfaA–E* genes, and the second group (EPA−) served as a control group that included the same strains but which were alternatively transformed with the *pfaA–D* genes without the *pfaE* gene and thus were unable to produce EPA. We evaluated the resistance of the EPA+ and EPA− lines against *t*-BHP as an agent for ROS and against antibiotics (ampicillin, kanamycin, chloramphenicol, and nalidixic acid) by measuring their minimum inhibitory concentration (MIC) values (Figure 3). In antibiotic resistance experiments, the MIC value of EPA+ K-12 cells was higher than that of EPA− K-12 cells, consistent with previous reports [51,55]. The same effects were found even if *ompC* and *ompF* mutants were used, indicating that the antibiotic resistance effect of EPA was not influenced by the Omp system. However, this effect was not detected in the AcrAB-TolC mutants, suggesting that the AcrAB-TolC drug efflux system is required for the action of EPA on antibiotic resistance. In the antioxidative effect experiments, all mutants exhibited the same levels of resistance for *t*-BHP as was seen in wild-type K-12 cells, except for *marA* mutant cells. These results suggest that although the AcrAB-TolC complex system is not involved in the antioxidative effect of EPA, MarA plays a role in this effect through its protein regulatory function. Considering that the AcrAB-TolC system is widely distributed in Gram-negative bacteria including EPA-producing bacteria, similar antibiotic resistance effects of LC-PUFA would be observed also in bacteria that natively produce LC-PUFAs.

### 3.4. Commercial Production and Use of LC-PUFAs

LC-PUFAs such as ARA, EPA, and DHA represent essential nutritional factors for humans. In particular, the *n*-3 LC-PUFAs EPA and DHA are associated with the suppression of various diseases such as cardiovascular health [60], Alzheimer’s disease [61], allergic diseases [62,63] and cancer [64,65], and therefore many countries recommend daily EPA and DHA intakes of 0.3–0.5 g [66]. The main source of EPA and DHA in the human diet is sea-fish oil. However, there is a limit to the amount of fish that can be sustainably harvested from available resources. Therefore, readily available and sustainable alternatives in place of fish oil are required.

Bacteria and eukaryotic microorganisms have been regarded as a promising source of LC-PUFAs. The benefits to using microorganisms include a stable supply of LC-PUFAs and the fact that microbial oils are not associated with marine pollution, fish odor, or fish allergies. Currently, some LC-PUFAs are produced industrially using eukaryotic microorganisms in the US and in EU countries. However, LC-PUFA-producing bacteria have not generally been utilized commercially because of their low LC-PUFA productivity.

In comparison, photosynthetic microalgae such as *Nannochloropsis oculata* (*N. oculata*) and *Phaeodactylum tricornutum* (*P. tricornutum*) accumulate EPA in triacylglycerols (TAGs) and EPAs extracted from *N. oculata* are now commercially available [67]. The levels of EPA produced by the genetically engineered *N. oculata* ST-6 represent 38%–39% of TFAs under optimal conditions and 2%–3% of its dry cell weight (DCW) is occupied by EPA [68]. For *P. tricornutum* UTEX 640, EPA represents 31% of TFAs and 5% of DCW [69]. On the other hand, the soil fungus *Mortierella alpina* 1S-4 [70], which was first isolated as a remarkably ARA-accumulating organism [71], and the budding yeast *Saccharomyces cerevisiae* FS01699 [72] are now being utilized to increase EPA productivity through genetic modifications. In these microorganisms, LC-PUFAs are most likely produced by the common combination of oxygen-dependent desaturation and fatty acid elongation but not by PUFA synthase.

In addition, various kinds of heterotrophic microalgae such as *Aurantiochytrium limacinum* SR21 [73], thraustochytrid strain 12B [74], *Ulkenia* sp., and *Crypthecodinium cohnii* (*C. cohnii*) [67] are known as good DHA sources. DHA from *Schizochytrium* sp., *Ulkenia* sp., and *C. cohnii* is commercially purified; these exhibit high levels of DHA contents reported as 45%–52% of TFAs and 20%–24% of DCW for *Schizochytrium* sp. [75], 10%–23% of TFAs and 5% of DCW for *Ulkenia* sp. [76], and 53%–57% of TFAs and 5%–6% of DCW for *C. cohnii* ATCC 30556 [77]. It should be noted that some of these microalgae also contain homologs of PUFA synthase [47,78,79] (Figure 1).

As a potential substitute for EPA and DHA supplied in TAG forms, the phospholipid (PL) forms of EPA and DHA derived from Antarctic *krill* (*Euphausia superba*) oil have attracted considerable amounts of attention because these are considered to be more effective as a nutritional source for human health than are the TAG forms of EPA and DHA that are rich in fish oil [80,81,82]. However, the use of *krill* as a sustainably available resource of LC-PUFAs is limited. Notably, the TAG accumulating DHA and ARA forms can be easily converted *in vivo* to the PL form in cells of the DHA-producing thraustochytrid strain 12B [83] and in an ARA-producing fungus [84], respectively, by the glucose starvation culture technique in which the cells are first cultivated in high concentrations of glucose (or other carbon sources) to accumulate LC-PUFA-rich TAG followed by cultivation in medium supplemented with no glucose. This technique is considered promising to produce various kinds of LC-PUFA such as the PL form.

Overall, the production of LC-PUFAs using microorganisms is considered to be both a feasible and a promising technology. DHA-enriched milk from cattle [85], the muscle tissue of pigs [86], and the egg yolk of layers (hens) [87] are produced using microbial DHA as feedstuffs. The only task to be overcome for the effective production of microbial LC-PUFAs is the reduction of its cost. Furthermore, considering that the lipid and fatty acid compositions of heterotrophic microorganisms such as *Schizochytrium* and *Aurantiochytrium*, which are capable of producing LC-PUFAs via PUFA synthase, are much simpler than those of fish and *krill*, these microorganisms might be more suitable for the production of highly purified LC-PUFAs as a fine chemical.

## 4. Evolution of C31:9 and LC-PUFA-Producing Bacteria

### 4.1. Possible Role of LC-HCs in Anaerobic Environments

EPA and DHA are often called “marine lipids” owing to their preferential distribution in marine environments [52]. However, recent studies show that the *pfa* genes encoding the PUFA synthase proteins and their homologs are also widely distributed in terrestrial bacteria including aerobic bacteria such as *Rhodococcus* spp. and strictly anaerobic bacteria including *Geobacter* and *Desulfobacterium* spp. [16].

Furthermore, some bacteria classified as obligate anaerobes are capable of synthesizing ROS scavenging enzymes such as superoxide dismutases, catalases [88,89,90], superoxide reductases, and peroxidases [91,92,93]. The NADH:rubredoxin oxidoreductase (NORO) complex is found in *Clostridium* species and an oxygen reductase is present in *Desulfovibrio vulgaris* to reduce cytoplasmic oxygen, NO, and ROS [94]. *Geobacter sulfurreducens* can also grow using oxygen as a terminal electron acceptor and can tolerate long-term exposure to oxygen [95]. These findings suggest that the ability to tolerate oxygen exposure is an important feature for the survival of these anaerobic bacteria when they are transiently exposed to aerobic circumstances [95,96].

The metal-reducing obligate anaerobic bacterium *G.* *bemidjiensis* Bem^T^, which was isolated from a terrestrial aquifer, carries the *pfaD*, *A*, *B*, *C*, *oleA*, *B*, *C*, *D* and *pfaE* genes in tandem (Figure 1). Although the metabolites produced by these *pfa* and *ole* gene products in the anaerobic bacterium are largely unknown, *G.* *bemidjiensis* Bem^T^ has been shown to produce a very long chain polyunsaturated hydrocarbon hentriacontanonaene (C31:9) [97]. It is speculated that PUFA synthases are involved in the production of intermediate polyunsaturated fatty acids and that these are subsequently transformed into C31:9 by the Ole proteins [12,13]. Although no direct evidence has been available, C31:9 and related LC-HCs of obligate anaerobic bacteria might be responsible for providing an antioxidative function such as performed by the LC-PUFAs in facultatively anaerobic bacteria such as *Shewanella* species when oxygen or exogenous ROS are encountered [50,51,52].

**Figure 4:**
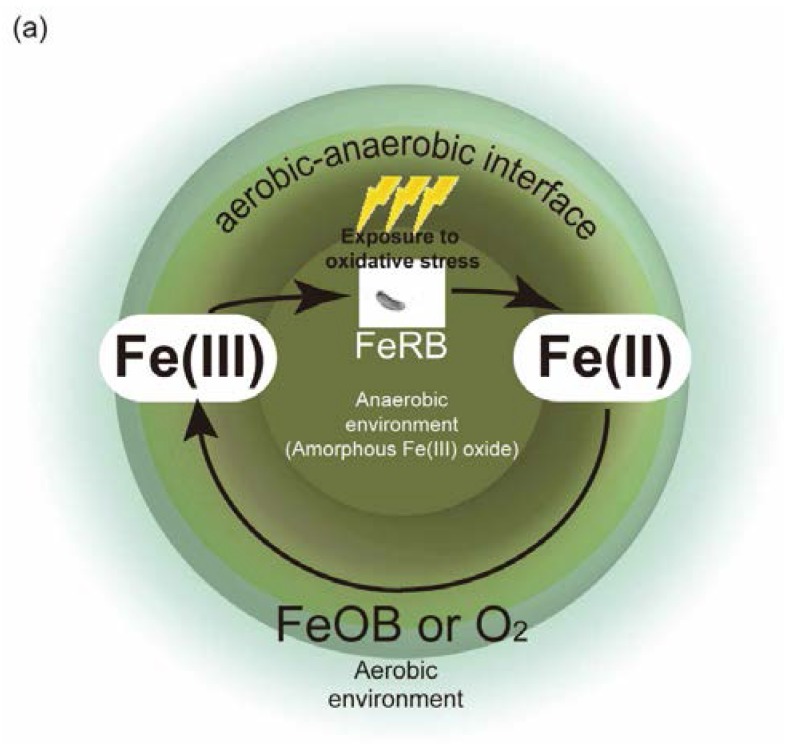

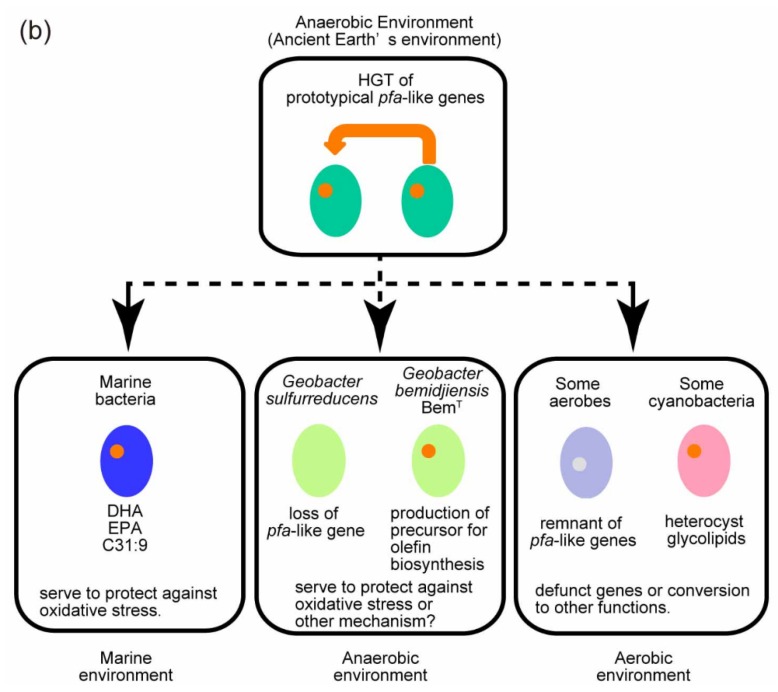
(**a**) Schematic diagram of possible functions of LC-PUFAs and LC-HCs. The conceptual model of the aerobic-anaerobic interface is taken from Roden *et al.* [101]. Metal-reducing bacteria (FeRB) harboring the *pfa*-like and *ole* genes (for example, *Geobacter bemidjiensis* Bem^T^) are assumed to produce LC-PUFAs and/or LC-HCs. Those genes might have been evolutionarily obtained (via HGT?) or conserved as descendant genes from ancestral bacteria that harbored the *pfa*-like and *ole* genes. FeRBs utilize Fe(III) (amorphous Fe(III) oxide) as the terminal electron acceptor and reduce it to Fe(II) under an anaerobic environment. Fe(II), in turn, is oxidized by iron-oxidizing bacteria (FeOB) or oxygen (O_2_). The environment becomes more aerobic as it is separated from the center of the circle (anaerobic environment; brown circle). FeRBs are exposed to oxidative stress at the aerobic-anaerobic interface, which might be potentially alleviated by LC-PUFA and/or LC-HCs. (**b**) A possible route for conservation of the *pfa*-like gene in anaerobic bacteria. This conceptual model, though speculative, shows the possibility of the *pfa*-like gene being harbored in anaerobic bacterium capable of producing LC-PUFA and/or LC-HCs. For comparison, possible routes of *pfa*-like gene conservation in marine and aerobic bacteria are also depicted in this figure.

Finally, manganese and iron reduction in sediments are known to play important roles in the biogeochemical cycle of many elements including carbon, sulfur, phosphorus, and several trace elements [98]. The process by which microorganisms couple the reduction of manganese or iron oxides to the oxidation of organic compounds (or H_2_) is often referred to as dissimilatory Fe(III) or Mn(IV) reduction [99]. Microbial manganese and iron reduction coupled to organic matter oxidation requires the direct contact of bacteria with the oxide surface [100]. Iron-reducing bacteria are assumed to exist in the aerobic-anaerobic interface where bacterial Fe(II) oxidation and Fe(III) reduction activities are likely to have coexisted within the same horizon [101]. Fe(II) is also subject to spontaneous chemical oxidation by oxygen and Mn(IV) oxides at circumneutral pH [102,103,104,105]. A conceptual model of this interface is depicted in Figure 4a. *G. bemidjiensis* Bem^T^ for example is assumed to exist in the aerobic-anaerobic interface in sediments, where the bacterium could utilize Fe(III) as the terminal electron acceptor, and where it, in turn, is likely to be exposed to oxidative stress. One possible explanation regarding why *G. bemidjiensis* Bem^T^ possesses the *ole* and *pfa* genes might be that this bacterium utilizes LC-HCs to protect itself against the occasional incursion of oxidative stress in the aerobic-anaerobic interface. Specifically, the *pfa*-like and *ole* genes might be evolutionarily obtained and conserved to survive under such an environment. Although this notion is still speculative and studies on the relationship between oxidative stress and LC-PUFAs and LC-HCs in anaerobic bacteria remain rudimentary, it is likely that further examination of the antioxidative functions of LC-PUFAs and LC-HCs in anaerobic bacteria might contribute to the elucidation of the evolutionary process of *pfa*-like genes.

### 4.2. Evolution of pfa-Like Genes

Shulse and Allen argue that the patchy distribution of secondary lipid pathways among bacteria and eukaryotic phyla might be attributed to horizontal gene transfer (HGT) [16]. We discuss here one possibility of HGT of the *pfa* and *ole* genes in bacteria from the viewpoint of the defense mechanisms against oxidative stress in surface and subsurface environments (Figure 4b).

HGT of the *pfa*-like and *ole*-like gene prototypes is assumed to have occurred frequently in the ancient Earth environment among a wide variety of bacterial phyla [15]. An example of HGT of the *pfa* gene cluster is reported to be present in one strain of *Vibrio* spp. but absent in many closely related *Vibrio* genomes [15]. Comparing the *pfa* gene region of *Vibrio splendidus* 12B01 with corresponding and flanking regions of the genomes of other *Vibrio* species, it was concluded that the *pfa* gene cluster had clearly been inserted into a genome island [15]. Polz *et al.* pointed out that the horizontal acquisition of genes in bacteria is frequently observed within particular functional categories, which include many surface structures such as O-antigen or membrane-spanning transporters as well as secondary metabolite production/modification [106]. They hypothesized that the horizontally-acquired genes might ensure resistance to rapidly changing biological factors such as immune defenses or viral and protozoan predation and confer competition interference and predation resistance [106]. Since PKS systems are regarded as secondary-metabolite gene clusters [107], it is likely that some bacteria harboring the *pfa*-like genes have acquired them by HGT. A prototype of the *pfa*-like and *ole*-like genes, which might have been similar to the contemporary *pfa*-like and *ole*-like genes, might have existed in primeval anaerobic bacteria that are assumed to have synthesized substances similar to LC-PUFAs and LC-HCs. Some facultative and strict anaerobic bacteria such as *Colwellia psychrerythraea*, *S. oneidensis*, and *G. bemidjiensis* Bem^T^ might have continued to maintain the *pfa*-like gene prototype, which might have rendered them a slight advantage over other bacteria in their environmental niche; for example, conferring a protective role against oxidative damage as the atmospheric O_2_ concentration increased. On the other hand, some strict and facultative anaerobic and aerobic bacteria might not have acquired them from the start or might have lost them during the course of evolution (Figure 4b). It is possible that the *pfa* genes found in *Clostridium thermocellum*, *Desulfobacterium* *autotrophicum*, and *Rhodococcus erythropolis* might be remnant genes [12]. Furthermore, given that no aerobic bacteria that synthesize LC-PUFAs and/or LC-HCs by the Pfa and Ole systems have yet been reported, almost all aerobic bacteria might have lost the activity to synthesize these compounds via the anaerobic pathway.

Some cyanobacteria, which are oxygenic and harbor a *pfa*-like gene, *hglE* [108,109], might instead have started to synthesize other specialized substances such as heterocyst glycolipids at the commencement of nitrogen fixation. Heterocyst glycolipids serve to protect the oxygen-sensitive nitrogenase enzyme in such specialized cells, termed heterocysts [110]. The losses of the *pfa*-like genes and the ability to synthesize LC-PUFA and/or LC-HC might be frequently observed in aerobic bacteria as they likely were substituted for other types of oxidative stress defense mechanisms such as catalase and superoxide dismutase.

## 5. Conclusions and Perspectives

The *pfa* genes with their diverse cluster organization and domain structure are widely distributed among bacteria and eukaryotic algae. These bacteria include those categorized as marine and terrestrial bacteria, gram-negative and gram-positive bacteria, strictly and facultatively anaerobic, and strictly aerobic bacteria. In marine environments, the *pfa* genes have been found in algae that belong to two different heterokont and haptophyte groups (phyla). The *pfa* genes are considered to encode the PUFA synthase responsible for LC-PUFA biosynthesis via the so-called anaerobic pathway in bacteria and algae. However, the production of LC-PUFAs has been confirmed only in limited numbers of isolated bacteria and algae and the synthesized LC-PUFA molecular species appear to depend on the type of *pfa* cluster. For example, in most bacteria, the type A, type B, and type D cluster organizations are involved in the synthesis of EPA, DHA, and ARA, respectively; with the exception of some myxobacterial species that exhibit type L organization yet also have the ability to produce ARA, EPA, and DHA. Notably, the AGPAT functional domain is accommodated only in the type L *pfa* gene cluster (*pfa3*, see Figure 1) of myxobacteria. Eukaryotic algae that carry the type E organization principally produce DHA.

The full set of *pfa* genes encoding the PUFA synthase is believed to be responsible primarily for the synthesis of LC-PUFAs, the mechanism of which can be relatively easily deduced from the functional domain structure of the individual Pfa proteins as these domain structures are quite similar to those of the known type I and type II FASs and those of the various types of PKSs. In this article, we first proposed mechanisms for the chain-termination of LC-PUFA biosynthesis in bacteria [111]. The most probable chain-termination in the LC-PUFA synthesis process likely occurs by their release from the ACPs accommodated in the PfaA protein by the thioesterase encoded by the newly designated *orf6* gene, which is present proximately upstream the *pfaA* gene of the type A and type B clusters in some bacteria. In addition, in the *Schizochytrium* system a thioesterase domain integrated within the PUFA synthase has been reported to participate in its LC-PUFA synthesis [111]. Further characterization of the Orf6 protein is required to specify its involvement in LC-PUFA synthesis.

The *in situ* role of the *pfa* genes is still unknown in almost all organisms. One of the primary and common functions of the *pfa* genes might be the synthesis of medium chain PUFAs, which can be used as a substrate of the Ole proteins to generate LC-HCs such as C31:9. This is suggested because almost all isolated bacteria that synthesize LC-PUFAs produce C31:9 whereas some bacteria including strict anaerobic bacteria are capable of producing C31:9 but not LC-PUFAs. Therefore, the *pfa* genes are likely to primarily synthesize C31:9 or other types of polyunsaturated HCs. Detailed analysis of the products generated by the Pfa proteins in *pfa*-carrying bacteria could be expected to allow a more thorough understanding of the relationship between the *pfa* genes and their *in situ* products. Similarly, the recent analysis of the structure of the tandem ACP domains existing within PfaA by the small-angle X-ray scattering method suggested that the structural flexibility of the tandem ACP contributes in an additive and parallel manner toward LC-PUFA production. The continued application of additional new methods using purified PUFA synthase and individual Pfa proteins is necessary to clarify their *in vivo* functions.

The global but patchy ecological distributions of LC-PUFA-producing bacteria and eukaryotes that carry *pfa* genes are both interesting and informative. In bacteria, *pfa* genes but not LC-PUFAs are commonly distributed in both strict and anaerobic bacteria and in aerobic bacteria as well. In the latter, no LC-PUFA products generated by the *pfa* genes have been reported, whereas some strictly anaerobic bacteria produce C31:9 using both the Pfa and Ole protein systems. Considering these findings and the antioxidative function of LC-PUFAs in bacteria, we assumed the evolutionary movement of *pfa* genes via HGT from hypothetical ancestral anaerobic bacteria to the existing strictly and facultatively anaerobic bacteria and aerobic bacteria that carry *pfa* genes. Of these, the aerobic bacteria would likely not be required to produce LC-PUFAs as a membrane phospholipid component or as medium chain PUFAs as a precursor of C31:9 or other polyunsaturated HCs via Pfa and Ole to protect themselves against ambient oxygen or exogenous ROS. This is because oxic challenges to aerobic bacteria always exceed their capacity to reduce the toxicity of oxygen by LC-PUFAs or LC-HCs and they are capable of synthesizing any PUFAs via the aerobic pathway (by aerobic desaturation and elongation of the fatty acids) even if these compounds are needed.

A recently obtained realization of the physiological role of LC-PUFAs in bacteria is that of their participation in specific membrane functions rather than in the adjustment of the physical fluidity of the whole cell membrane. The newly-understood functions of LC-PUFAs, which have been specified using EPA-producing bacteria and their EPA-deficient mutants as well as EPA-producing *E. coli* recombinants carrying *pfa* genes, include an antioxidative activity against exogenous ROS, membrane efflux activity toward antibiotics, and microdomain formation responsible for cell division at low temperatures and/or high pressures. In addition to the techniques describe above, artificial membrane systems reconstituted with selected purified membrane proteins and phospholipids containing LC-PUFAs as their acyl components might also serve to further elucidate the physiological roles of LC-PUFAs.

In addition to their functions in bacteria and eukaryotic microorganisms, LC-PUFAs have considerable nutritional and pharmaceutical value as well. A better understanding of the specifics of LC-PUFA production and function in specific microorganisms will likely facilitate the development of cost-effective and sustainable commercial production protocols of LC-PUFAs, as well as providing clues toward the potential pharmacologic utilization of these compounds in novel areas as further potentially unexpected functions are discovered.
